# The Treatment of Chorea

**Published:** 1893-01-21

**Authors:** 


### The Hospital Clinic.

[*The Editor will be glad to receive offers of co-operation and contributions from members of the profession. All letters should be addressed to* THE EDITOR, THE LODGE, PORCHESTER SQUARE, LONDON, W.]

### THE DEVON AND EXETER HOSPITAL.

### The Treatment of Chorea.

It is sometimes said that chorea is less common in agricultural districts than in large centres of population, and this appears to be founded on the idea that fright is a frequent cause—fright being presumably more common in crowds. But although fright is certainly seldom alleged here as the cause of the cases, the admissions for chorea at the Devon and Exeter Hospital are proportionately greater than in the London hospitals, and consequently a few words on the modes of treatment adopted may be of interest.

The first point in the treatment is that the child should be taken into the hospital and put to bed, unless, indeed, the movements are extremely slight and of recent origin, in which case she (for, as is usually the case elsewhere, we find female children mostly affected) may be treated as an out-patient.

A second point is to isolate the patient as much as possible. In this hospital there are a number of separation wards, and a very bad case is at once placed in one of them, whilst the less serious are treated in a general ward, in a corner bed if possible, and with screens placed round and kept there day and night so as to isolate them, whilst still keeping them under close observation of the nurse.

In cases where the movements are very violent and extensive, a mattress is hung up by the side of the wall, and a thick pillow or bolster is tied over the head of the bed.

A third most important point is the diet; the child is fed regularly with at first milk diet, then fish, and finally ordinary full meat diet, in accordance with the severity of the disease and the rate of improvement. It is difficult, perhaps, to estimate the value of the regular feeding and the nourishing diet; but when we consider that many of the children come from very poor homes, and are from the severity of their movements unable to feed themselves, there can be no doubt that they have a considerable influence on the result of the treatment.

So much for three general rules of treatment: hospital, bed, and good, regular, nourishing diet.

It has long been a recognised fact here that chorea may be entirely due to intestinal worms, and in searching the past records nearly every case is found to have been at once treated with anthelmintics. The success that attended this treatment was often remarkable, five grains of santonin with large doses of castor oil often bringing away a large number of worms, with the result that in a few days, or with a repetition of the santonin, the child lost all its movements. The treatment is still carried out with success, though not in quite such a routine manner as was previously the case, and often when there has been no previous history of worms, a dose of santonin proves their presence, and places the child in the way to recovery.

The cases treated in this hospital, though a fatal case is exceptional, are many of them of a severe type, and perhaps, in considering the treatment by drugs, it will be well to make a division into the acute and chronic cases.

In the acute cases many drugs have been tried, and the conclusion come to with regard to arsenic is interesting. Some of the physicians have given, and in some cases still continue to give arsenic in increasing doses, beginning with liquor arsenicalis, 2 minims three times a day, in a child of ten years of age, and increasing it up to 10 or even 15 minims. But the success generally accorded to this drug has not been met with here; it seems that it does not shorten the duration of the attack much, if at all, and when it does good it often does so by first increasing the severity of the attack.

One member of the staff has almost entirely given up the use of arsenic, and his routine treatment is to give half an ounce of peppermint-water three times a day, isolate the patient, and feed carefully. The experience afforded by one very severe case in this respect is worthy of record. It was the case of a girl, aged 19, with sudden onset due to worry; she came in with moderate movements, which rapidly became worse after admission, and though arsenic, bromide, large doses of belladonna, and ergot were tried, no drugs were of use. Unable to take food, delirious, and apparently dying, all medicine was stopped, and as a last resource 5 minims of morphia were injected hypodermically, followed shortly by another 4 minims. She had a quiet night, improved rapidly, and was convalescent in a fortnight.

Bromide and chloral have both been given, but neither have met with such marked success as to warrant a general use of them, while ergot, which was much given at one time, is not often used now.

Many of the cases, however, met with in this hospital are accompanied by anæmia and a low state of nutrition, often induced by want of proper food outside. In these circumstances we have two drugs that have been used with marked success, cod liver oil and iron. It is wonderful to note how rapidly a child improves on cod liver oil in teaspoonful doses three times a day, given by itself or in the more palatable form of Scott's emulsion. In other cases, three grains of the citrate of iron and ammonia cure both the anæmia and the chorea. A prescription used by one of the staff consists of camphor 2 grains, cod liver oil 1 dram, hypophosphite of soda 5 grains, sal volatile 15 minims, and water to 1 ounce. Several other drugs have been tried, as, for example, zinc and antipyrin, but not to so great an extent as to justify an opinion being formed on their merits, while in cases of adults or young females, an effervescing mixture of valerian has been given with good effect. Exalgin has not been tried.

Chloroform has not been used except in the very bad cases, and then rather as a last resource; but feeding by the rectum with nutrient enemata, when the patient is prevented by the violence of her movements from taking food, has been found very useful.

In the more chronic cases the effect of santonin is still marked, as the presence of worms has often not been suspected; then, in addition to the tonic treatment, arsenic seems to have good effect. It is given in large doses and continuously, sometimes until its effect is plainly visible on the skin, and in many chronic cases, unlike the acute, the result has been very good. In these cases, too, the careful diet, with bed and isolation are of great use.

As a general result, then, of the treatment of a large number of cases, we find that in acute cases arsenic does not hold the place usually assigned to it; that in chronic cases it is decidedly useful, but that those cases which have been treated on rational principles, with especial regard to diet and the removal of exciting causes, and with no medicine at all, or with cod liver oil and iron, have done extremely well.

We find, too, that intestinal irritation is a very potent cause of chorea in children, as evidenced both by their improvement when treated by anthelmintics and even when no worms are discovered, by their improvement under proper feeding.

With regard to the duration of the cases under observation, it certainly is not so long as that generally taught in the text books. Gower's, for example, gives the limit as from six weeks to six months, and seldom less than the latter, whereas few of the cases here reach the limit of six months, and fewer still go on for twelve or more months. How much of this is due to treatment, and how much to the greater opportunities afforded by a provincial hospital and its surroundings, is difficult to decide.

It only remains to say a few words on the after treatment of chorea, and the liability to relapse.

Relapses are not very common when once the patient has been thoroughly cured, and no patient is sent out if it is possible to keep her, until the movements have quite ceased, and the general health and mental condition have reached their normal state.

The child is then, if feasible, sent either to a convalescent home or to friends in the country, a most important point, as it prevents her returning at once to the surroundings which have often been instrumental in inducing the attack. She carries with her a supply of tonic medicine or cod liver oil, which she continues to take for some weeks, and at the end of this time she has generally lost her chorea for ever.

